# Proof-of-principle experiment for laser-driven cold neutron source

**DOI:** 10.1038/s41598-020-77086-y

**Published:** 2020-11-19

**Authors:** S. R. Mirfayzi, A. Yogo, Z. Lan, T. Ishimoto, A. Iwamoto, M. Nagata, M. Nakai, Y. Arikawa, Y. Abe, D. Golovin, Y. Honoki, T. Mori, K. Okamoto, S. Shokita, D. Neely, S. Fujioka, K. Mima, H. Nishimura, S. Kar, R. Kodama

**Affiliations:** 1 Institute of Laser Engineering, Osaka University, Suita, Osaka 565-0871 Japan; 2 Blackett Laboratory, Imperial College, London, SW7 2AZ UK; 3 National Institute for Fusion Science, Toki City, Gifu 509-5202 Japan; 4 Rutherford Appleton Laboratory, Central Laser Facility, Didcot, Oxfordshire OX11 0QX UK; 5 The Graduate School for Creation of New Photonics Industries, Hamamatsu, 431-1202 Japan; 6 Faculty of Engineering, Fukui University of Technology, Fukui, 910-8505 Japan; 7 Centre for Plasma Physics (CPP), Queen’s University of Belfast, Belfast, BT71NN UK

**Keywords:** Plasma-based accelerators, Laser-produced plasmas

## Abstract

The scientific and technical advances continue to support novel discoveries by allowing scientists to acquire new insights into the structure and properties of matter using new tools and sources. Notably, neutrons are among the most valuable sources in providing such a capability. At the Institute of Laser Engineering, Osaka, the first steps are taken towards the development of a table-top laser-driven neutron source, capable of producing a wide range of energies with high brightness and temporal resolution. By employing a pure hydrogen moderator, maintained at cryogenic temperature, a cold neutron ($\le 25\hbox { meV}$) flux of $\sim 2\times 10^3\hbox { n/cm}^2$/pulse was measured at the proximity of the moderator exit surface. The beam duration of hundreds of ns to tens of $\upmu \hbox {s}$ is evaluated for neutron energies ranging from 100s keV down to meV via Monte-Carlo techniques. Presently, with the upcoming J-EPoCH high repetition rate laser at Osaka University, a cold neutron flux in orders of $\sim 1\times 10^{9}\hbox { n/cm}^2/\hbox {s}$ is expected to be delivered at the moderator in a compact beamline.

## Introduction

**Figure 1:**
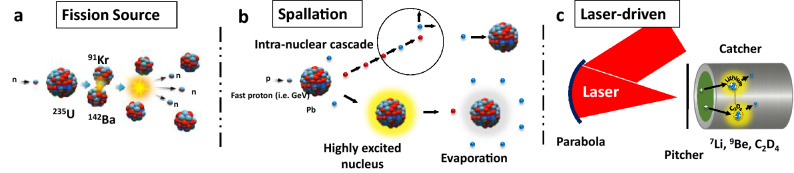
Illustration of neutron generation. Schematics is showing the (**a**) Fission, (**b**) spallation and (**c**) laser-driven pitcher-catcher schemes. Nuclear reactors produce neutrons by splitting of $^{235}$U nuclei in process known as fission, whereas the spallation reactions employ high energy ions to bombard a high Z target. On the other hand, the laser based schemes generate neutrons via light ion fusion and nuclear reactions in a converter target, known as catcher, such as $^2_1d(d,n)^3_2He$ and $^7Li(p,n)^7Be$ driven by multi-MeV ions produced from laser-driven thin foils known as pitcher. While fission reactors are continues by their nature, spallation and laser-driven sources are pulsed owing to the ultra-short duration of the driving ion beam, and that is in orders of μs and ps respectively.

The pioneering works on radiation developments in the nineteenth century, and later the discovery of the neutron in 1932^1^ enabled emergence of new applications in material science and archaeology^2^, chemistry^3^, radiography^4^, and biology^5^. As of today, neutrons for research are primarily produced using nuclear reactors and spallation sources. In the nuclear reactors, the absorption of a single neutron in a fissile material such as $^{235}$U splits the nucleus into small fragments and liberates multiple neutrons in a chain process known as fission, as shown in Fig. 1a. While the sources of this type are continuous with some exceptions^6^, many applications such as neutron scattering required a short burst duration, hence, the quest for a new type of sources resulted in the development of spallation facilities. The spallation is referring to the interaction of energetic particles (e.g. proton and deuteron) with a high-Z element. The collision leads to the generation of a highly excited nucleus and series of intra-nuclear reactions, releasing neutrons as shown in Fig. 1b. An important difference between the fission and spallation mechanism is the number of useful neutrons produced per event, for instance, with the thermal fission of $^{235}$U, a portion of the neutrons are absorbed by the fuel to sustain the chain reaction, whereas in spallation that is not the case, thus a higher flux of neutrons at the sample is available. Another notable advantage of spallation sources is the neutron burst duration, which is defined by the length of the driving ion bunch, and it is usually around tens of $\mu$s compared to ms in reactors^7–9^. Moreover, with the fission sources, the nuclear wastes and their proliferation are among the other issues that are always being debated. Despite many of the benefits, due to the high costs and lack of their availability, new compact arrangements known as Compact Accelerator-driven Neutron Sources (CANS) appeared to fill the gap by providing the capabilities for a wider range of research and multidisciplinary applications, while operating at moderated power and smaller scales^10,11^.

In the pursuit of fusion sources and the construction of the first neodymium-doped glass (ND: glass) laser by Lawrence Livermore National Laboratory (LLNL) in 1972 and the concept of Chirped Pulse Amplification (CPA) in 1985^12^, ion beams by lasers became a reality^13–15^. The interaction of lasers at relativistic intensities ($\ge 10^{18} \text{ W } \text{ cm}^{-2}$) with a (100s nm to 10s $\upmu$m) thick target produces a large number of fast electrons. These electrons approximately have a similar energy to the ponderomotive potential of the laser at its focus. With energies of above tens of keV, they are continuously accelerated with a mean free path of 100s of μm. As they propagate through the target into the vacuum, a sheath field at the rear side is produced, leading to the creation of a strong driving force, resulting in ions being accelerated to tens of MeV^16^ in a scheme known as Target Normal Sheath Acceleration (TNSA) ^17^. Due to the high charge-to-mass ratio of hydrogen isotopes compared to heavier ions (e.g. carbons), they are accelerated more efficiently and in great abundance. The accelerated ions leave the target rear surface in a small angular divergence, decreasing to $10^\circ$ at high energies, which can be further improved using magnetic focusing devices^18–20^, micro-lens^21^, helical coil^22^ and specially shaped targets^23^.

As shown in Fig. 1c, with ultra-intense lasers, fast neutrons are produced in a scheme known as pitcher-catcher mechanism^24^, where the high energy ions from the target (the pitcher) are used to drive the light ion reactions (e.g. fusion, breakup/stripping) in a suitable converter material such as $^9Be$, $^7Li$, $C_2D_4$ (known as the catcher) at hundreds of picoseconds with high brightness^25–34^. The rapid progress in the advancement of table-top lasers^35–37^, made it possible to produce sources satisfying the requirement of CANS both in terms of cost and compactness. Consequently, as the CANS are up and running, a new type of source based on ultra-intense lasers is forthcoming.

The neutrons produced with the above reactions are usually at > 100 KeV energy range (known as fast) and are very penetrative. While the fast neutrons are useful to study nuclear structure, it is desirable to cool them down to lower energies using a moderator to exploit their potential even further. Therefore, two primary effects are taken into account when a moderator is developed: *(1)* neutron leakage and *(2)* the pulse duration. Typically, the purpose of a moderator is to cause a sufficient amount of energy loss to the incoming neutron energies. This is done via a series of elastic and inelastic collisions where energy exchange between the medium and the incoming neutrons is given as a function of the scattering angle. The materials are usually selected based on their moderating power (MP), given by the average logarithmic energy decrement and the macroscopic scattering cross-section. A higher MP does not necessarily guarantee a high moderation efficiency, this is because a good moderating ratio requires a smaller absorption cross-section, known as the ratio of scattering power over absorption. For this reason, hydrogenous based moderators (e.g. $H_2$, $CH_4$) are the most efficient. Finally, the pulse duration is expressed as the effective moderation length and the geometrical time-of-flight distance, therefore, it is crucial to keep the moderator size as small as possible.

Moderated neutrons are generally categorized in terms of energy with fast (>100 keV), epithermal (0.5 eV – 100 keV)^38^, thermal (25 meV)^38^, and cold reaching $\le$25 meV. The epithermal neutrons are of high interest for a wide range of applications related to condensed matter such as Deep Inelastic Neutron Scattering (DINS)^39^, Neutron Resonance Absorption (NRA)^40^, nucleosynthesis processes of astrophysical relevance^41^ and Boron Neutron Capture Therapy(BNCT)^42^. The thermal neutrons, on the other hand, provide important information on the complex process of molecular and atomic vibration. Furthermore, the wavelength of cold neutrons are comparable to the interatomic distances making them suitable for refractometry, small-angle scattering^43^, and imaging applications^4^.

In this paper, we report the progress towards the experimental demonstration of a cold neutron source produced by an ultraintense short-pulsed laser. By directing fast neutrons of above MeV in the orders of $10^9$ n/sr/pulse, into a cryogenically cooled $H_2$ moderator, a cold neutron flux of $\sim 2\times 10^3\hbox { n/cm}^2$/pulse was estimated at 20 cm reachable distance from the exit surface of the moderator using sets of $^3$He detectors. The results discussed in this paper demonstrate the first data obtained at the Institute of Laser Engineering (ILE), Osaka-Laser-driven neutron source (LDNS).

**Figure 2:**
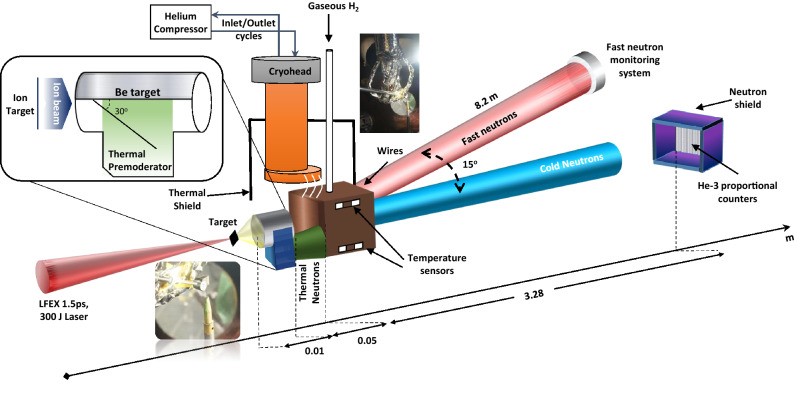
Osaka laser neutron source (LDNS). The cold neutron source is produced using cryogenically cold hydrogen moderator placed at $\sim \hbox {cm}$ from the catcher target. The system developed at ILE is using helium fluid to remove the heat from the cell via attached wires brought from the cryohead. The injected $H_2$ was cooled in less than a day, while having its temperature and pressure being checked using superconducting temperature sensors and flow/monitor controllers, respectively. A wing-shaped pre-moderator was added to catcher to thermalize the off-axis neutrons. After each shot, the fast neutron signal was recorded using the fast neutron detector sitting at 15$^\circ$, 8.2 m and He-3 proportional counters located on axis at 3.28 m.

**Figure 3:**
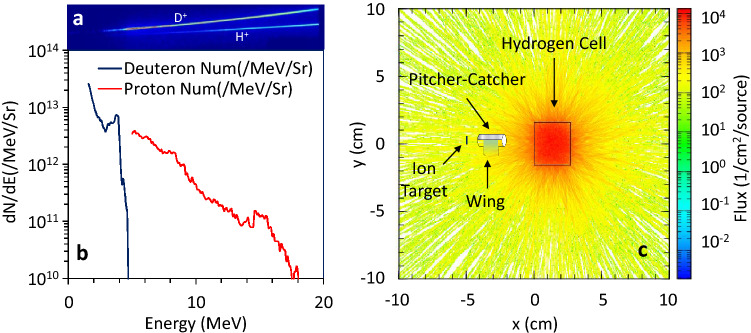
Ion-driver spectrum with corresponding neutron simulation. (**a**) Shows a typical proton and deuteron raw data obtained using $5\,\upmu \hbox {m}$ Au foil with TP, with respected spectra shown in (**b**). (**c**) shows the PHITS Monte-Carlo simulation performed for the cold neutrons ($\le 25\hbox { meV}$) in the hydrogen moderator, confirming an isotropic nature of the moderated neutrons.

## Results

The experiment was carried out at the Institute of Laser Engineering (ILE), Osaka using 1.2 ps beams of LFEX^44^, delivering total energy of 300 J on the target. A schematic of the experimental setup is shown in Fig. 2. The fast neutrons were produced by impinging the laser-driven accelerated ions from 5 μm thick $C_2D_4$ pitcher into a cm thick block of beryllium catcher placed at its proximity. The ion temperature for both proton and deuterium beams were recorded using Thomson Parabola (TP) prior to the neutron shots, as shown in Fig. 3a. The typical ion spectra exhibit an exponential decay for proton and deuteron signals, having a sharp cut off energy at $\sim 5\hbox { MeV}$ and $\sim 20\hbox { MeV}$ respectively, as shown in the Fig. 3b. The primary neutron producing mechanism is expected to occur via $^9Be(d,n)^{10}B$ and $^9Be(p,n)^9B$ reactions, with (*p*, *n*) reaction yielding a softer spectrum. Given the higher deuteron yield and reaction threshold starting at tens of keV, it is expected the $^9Be(d,n)^{10}B$ to be the main mechanism in generating neutrons.

Considering the advantage of neutron angular distribution with the $^9Be(d,n)^{10}B$ reaction for the measured deuteron energies^45–47^, an off-axis wing-shaped polyethylene pre-moderator was attached to the catcher, producing a thermal peak at room temperature. Additionally, in the future, a combination of reflectors will be employed to enhance the pre-moderated flux further. To deliver the shortest possible beam duration, the smallest possible wing dimensions ($10\hbox { mm }\times 6\hbox { mm}\times 5\hbox { mm}$) was considered. As for the main moderator in the cryogenic assembly, the $H_2$ gas was introduced via several stages into the moderator cell, while maintaining the correct pressure and temperature using flow/pressure controllers and helium cooling lines respectively. The hydrogen thickness along the beam axis was $\sim 27\hbox { mm}$ surrounded by copper reflectors of 2 mm thickness. Consequently, the cell was filled with liquid and further being cooled down to $\sim 11\hbox { K}$. The cell temperature was monitored using superconductor sensors placed at the outer surface of the moderator housing, nevertheless, it was expected that the actual temperature to be higher inside the cell. During the experiment, it was anticipated that the distribution of moderated neutrons, corresponds to the neutron energies in equilibrium, given by the temperature of the moderator assembly, with the peak of neutrons extended towards meV range^48^. Using PHITS Monte-Carlo code as shown in Fig. 3c, the neutron divergence is studied for energies below 25 meV in the hydrogen assembly, and the result confirms an isotropic distribution.

**Figure 4:**
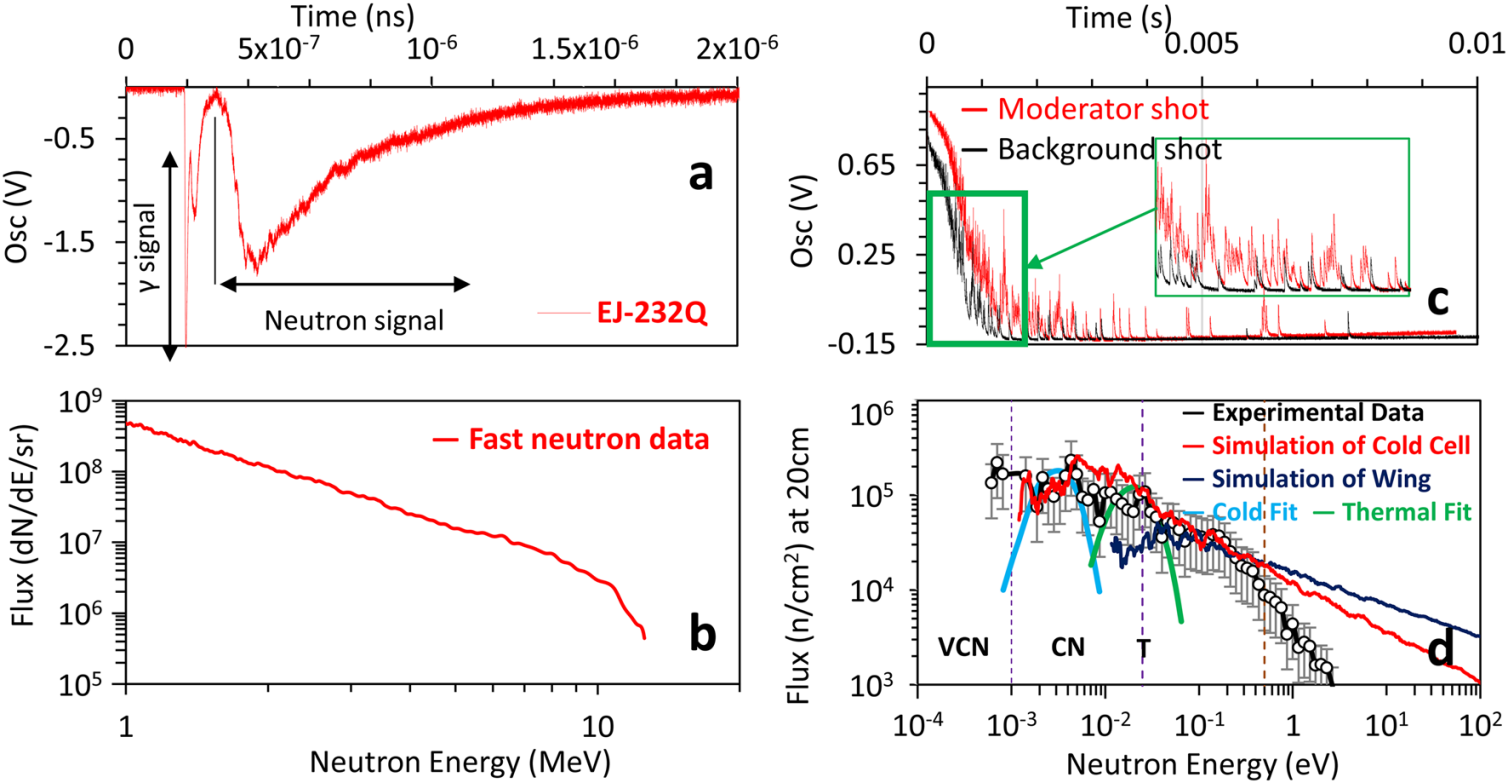
Experimental results. (**a**) is the raw signal recorded using EJ-232Q plastic scintillator and the corresponding spectrum shown in (**b**), which was calculated by taking into account the detector distance, efficiency and the transmission. (**c**) demonstrating the neutron spikes generated by nuclear reaction of $^3\hbox {He}(n,p)^3H$ in the proportional counter for a typical moderator shot (with hydrogen) and no moderator shot (without hydrogen). The background-subtracted data at 20 cm exit surface of the moderator showing a good agreement with the Monte-Carlo simulation results as shown in (**d**). The broadened peak of neutrons extended its tail reaching 0.8 meV and has been confirmed using two Maxwellian numerically calculated fits for the cold and thermal temperature. The navy blue line is showing the contribution of the wing moderator which is barely reaching the thermal peak.

During the neutron producing phase of the experiment, the fast neutron signal was characterized in a ToF arrangement by placing a plastic scintillator (EJ-232Q) at 8.2 m distance and 15$^\circ$ angle to have a clear line-of-sight for the incoming fast neutrons before the moderation takes place. Fig. 4a shows a typical raw trace obtained from the detector. During each shot, the γ signal produced by the laser-target interaction reaches the detectors simultaneously, therefore, it can be used to define the zero point. The neutron spectrum was then calculated by taking the distance, transmission and the detector efficiency^49^ into account. As seen, the γ trace exponentially decays to zero before the main neutron signal arrives, makes it very convenient to be extracted from the main neutron signal. Over several dedicated shots, the recorded neutrons yield fairly similar spectra with the highest flux in the $\sim \hbox {MeV}$ region ($\le 10^9$ n/sr/pulse), as shown in Fig. 4b.

Concurrent to the fast neutrons, the cold neutron spectra were recorded using He-3 proportional counters for the different cases of with-moderator and with no-moderator (background) shots. In order to remove the stray of scattered neutrons, the detectors were shielded by sandwiched plastic and cadmium layers, with an envelope left open for the on-axis neutrons to go through. Figure 4c is showing the neutron spikes recorded by the reaction of $^3$He$(n,H)^3H$ and the corresponding avalanche process created by the interaction of neutrons with the He-3 detector tube. These detectors operate in a linear regime where the measured pulse height is directly proportional to the gas ionisation, therefore, by the interaction of the incoming neutron, the nucleus breaks up into a tritium and a proton, leading to the liberation of electrons. The electrons are then accelerated towards the cathode, creating a detection cloud through charge multiplication. The detector output is typically a current pulse, which later is converted into a measurable signal using a preamplifier and then fed into an oscilloscope. Each hit is represented by a spike, used to calculate the neutron energy based on its arrival time.

**Figure 5:**
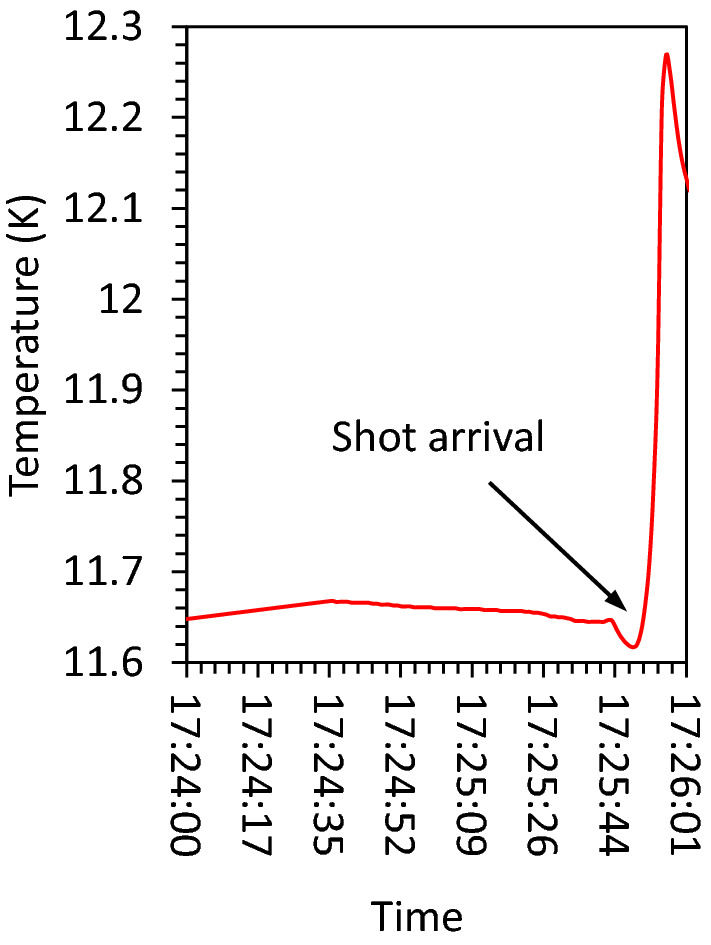
Moderator cell temperature. Showing the cell temperature measured using superconducting sensors. By the arrival of the neutrons, the cell temperature increased slightly, before stabilizing back into operating temperature in less than few minutes.

Similar to the fast neutron detector, the prompt γ signal used to define the zero point in the ToF calculations. As shown, the number of hits were significantly higher in a typical moderator shot (red line) when compared to the background case (black line). The corresponding background-subtracted ToF spectrum is shown in Fig. 4d, signifying a broadened peak of neutrons at around meV energy, as well as a small peak for the thermal neutrons primarily generated due to the wing moderator and the partially moderated portion of the neutrons. The spectrum obtained is showing a good agreement when compared to the corresponding Monte-Carlo simulation using the hydrogen cross-section at low temperature and employing the tmp cards of the code. The mismatch at epithermal energies is due to the early detector saturation by the γ-ray, lasting for several μs. The numerically calculated Maxwellian cold and thermal fits are additionally plotted to demonstrate the expected location of neutrons at the different temperatures. The simulation of wing moderator confirms that the neutron energies down to thermal range were only producible, which is mainly due to the insufficient thickness of the plastic. Figure 5, shows the cell temperature around ~ 11 K which was maintained before the shots.

Finally, by taking into account the detector efficiency and normalizing the signal with respect to the incident neutron flux measured on the plastic scintillator, the total cold neutron ($\le$ 25 meV) flux of $\sim 2\times 10^3\hbox { n/cm}^2$/pulse, and thermal neutron flux of $\sim 5\times 10^3\hbox { n/cm}^2$/pulse at a most reachable distance of 20 cm from the exit surface of the moderator were measured.

## Discussion

The experiment was designed to assess and demonstrate the feasibility of driving intense bursts of cold neutrons with short pulse lasers. By placing the catcher target inside a moderator and utilizing a good shield, one could produce multiple beams of neutrons at various energies in different directions. Currently, there is a continuing effort to improve the neutron flux by optimizing the moderator, adding reflectors and neutron guides as well as coupling a higher input neutron fluxes by taking advantage of the ongoing developments in laser-driven ion acceleration^50–52^. One of the benefits of laser-driven ion sources is their potential to produce softer neutron energy spectra using nuclear reactions at lower energies. This is because the ions at hundreds of keV to several MeV are produced in abundance with the currently well-established ion acceleration methods. For instance, using near-threshold reaction^53^ via $^7Li(p,n)^7$Be in TNSA, one could potentially continue to produce a fast neutron temperature at around tens of keV, therefore a smaller moderator system is required, and that leads to a shorter beam duration.

**Figure 6:**
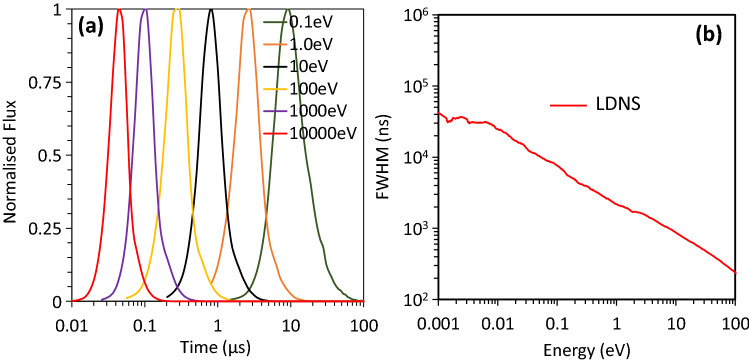
Temporal profile of neutrons. Simulated FWHM duration of laser-driven neutrons at 20 cm from the exit surface of the moderator with (**a**) is showing the selected neutron energy duration, and (**b**) is showing the the overall performance of Osaka LDNS.

In order to assess the temporal profile of the neutrons produced using the described LDNS, a Monte-Carlo simulation was performed for a 3 cm thick hydrogen moderator similar to the experimental setup. As shown in Fig. 6a, the pulse duration at $<1\hbox { eV}$ in our case was expected at $\sim$ 1–100 $\upmu \hbox {s}$, and it becomes significantly shorter (below 100 ns) at $\hbox {E}_n\gtrsim 10\hbox { eV}$. Under this condition, a relatively short distance between the moderator and sample would provide sufficient energy resolution useful for scattering experiments^7,9^. The pulse duration can be further enhanced by moderator poisoning or decoupling, at the cost of the overall system’s intensity. Figure 6 is demonstrating the moderating time performance of the overall system at different energies. Whilst laser-driven particle beams yet can be improved, the current experiment offers a pathway towards the implementation of adequate sources that can be placed at small laboratories in universities and industries.

With the current development of high repetition rate 10 PW lasers around the world, such as LIGHT beamline, GSI Helmholtzzentrum für Schwerionenforschung (GmbH) in Germany^54^, Extreme Photonics Applications Centre (EPAC)^55^ in UK, RAMI beamline in China^56^, and DiPOLE projects at Extreme Light Infrastructure - Nuclear Physics (ELI-NP)^57^, a peak neutron flux of $5\times 10^{22-24}\hbox {(n/cm}^2\hbox { s}$)^58^ is expected. Meanwhie, at ILE, Osaka, by employing the 100 Hz 1.5 kJ J-EPoCH table-top laser, a cold neutron flux in the orders of $\sim 1\times 10^{9}$ n/sr at the sample position is foreseen. The cold neutron obtained in this experiment is the first demonstration of its kind using lasers, and by devising the assembly, it would be desirable to conduct first experiments in which a laser-driven source is employed for scattering experiments. Furthermore, the current $H_2$ moderator experiment described can be used to deliver pre-moderated neutrons required for ultra-cold neutrons (UCN) generation by employing a few cm thick $SD_2$ moderator surrounding the cell, which can be used to perform astrophysical studies such as neutron life-time measurements using a compact light source.

In conclusion, a cold neutron source was demonstrated for the first time by utilizing a laser-driven fast neutron source coupled to a compact moderator. Due to the possibility of deploying samples in the proximity of the moderator, and thanks to the minimal radiation shielding requirement with the rising prospects of high repetition rate systems, laser-based sources are approaching a crucial stage in their development for neutron science and applications.

## Methods

### Experiment

The experiment was carried out using the LFEX laser at the Institute of Laser Engineering (ILE), Osaka, Japan. The laser pulse of $\sim$ 1.2 ps FWHM with the total energy of $\sim$ 300 J was focused on 5 $\upmu$m deuterated carbon $C_2D_4$ pitcher targets via an f/10 off-axis parabola, producing peak intensities of over $\sim 5\times 10^{18}~\text{ W } \text{ cm}^{-2}$. Initially, the laser-driven ion beam was characterized using Thompson Parabola (TP), and deuteron and protons of up to $\sim 5\hbox { MeV}$ and $\sim 20\hbox { MeV}$ were measured from the rear surface of the target respectively, indicating a Target Normal Sheath Acceleration (TNSA) acceleration mechanism. The accelerated ions were injected onto a cm thick $^{10}$Be converter to produce neutrons via $^9Be(d,n)^{10}B$ and $^9Be(p,n)^9B$ nuclear reactions. The neutron spectrum was diagnosed by the time of flight (ToF) technique using the neutron monitoring system, which included a fast scintillator detector (EJ232Q^49^ coupled to Hamamatsu R2083 PMT) to diagnose MeV neutrons, and He-3 proportional counters to measure cold neutrons, located at 8.2 m (15$^\circ$) and 3.28 m (on-axis) from the source, respectively. The detectors were shielded with lead and plastic layers to remove the stray of scattered neutrons, with He-3 detectors having additional cadmium sheets to remove the scattered neutrons coming at lower energy. The efficiency of the detectors was evaluated using $\eta =1-\hbox {exp}(-0.00482 P d \lambda )$, with *P* representing gas pressure in bar ($\sim 10\hbox { bar}$), *d* showing the tube diameter in mm and with neutron energy given in wavelength and unit of angstroms represented by $\lambda$.

### Modeling

The Particle and Heavy Ion Transport code system (PHITS)^59^, version 3.08 Monte-Carlo code was employed to simulate the transport of neutrons and cold neutron production in the moderator. The code incorporates JENDL-4 data libraries to calculate the particle lifetime through multiple events in the simulation. Using tallies, the user is able to output the needed information such as particle energy, flux and ToF across different XYZ planes.

## Data Availability

The datasets are available from the corresponding author upon reasonable request.
