# Author Correction: Proof-of-principle experiment for laser-driven cold neutron source

**DOI:** 10.1038/s41598-021-96558-3

**Published:** 2021-08-25

**Authors:** S. R. Mirfayzi, A. Yogo, Z. Lan, T. Ishimoto, A. Iwamoto, M. Nagata, M. Nakai, Y. Arikawa, Y. Abe, D. Golovin, Y. Honoki, T. Mori, K. Okamoto, S. Shokita, D. Neely, S. Fujioka, K. Mima, H. Nishimura, S. Kar, R. Kodama

**Affiliations:** 1 Institute of Laser Engineering, Osaka University, Suita, Osaka 565‑0871 Japan; 2 Blackett Laboratory, Imperial College, London, SW7 2AZ UK; 3 National Institute for Fusion Science, Toki City, Gifu 509‑5202 Japan; 4 Rutherford Appleton Laboratory, Central Laser Facility, Didcot, Oxfordshire OX11 0QX UK; 5 The Graduate School for Creation of New Photonics Industries, Hamamatsu, 431‑1202 Japan; 6 Faculty of Engineering, Fukui University of Technology, Fukui, 910‑8505 Japan; 7 Centre for Plasma Physics (CPP), Queen’s University of Belfast, Belfast, BT71NN UK

Correction to: *Scientific Reports* 10.1038/s41598-020-77086-y, published online 19 November 2020

The original version of this Article contained errors.

In Figure 3b, where

“Deutron”

now reads:

“Deuteron”

In addition, in Figure 4d, where the x-axis

“10^–4^, 10^–3^, 10^–2^, 10^–1^, 10, 10^2^, 10^3^”

now reads:

“10^–4^, 10^–3^, 10^–2^, 10^–1^, 1, 10^1^, 10^2^”

The original Figures 3 and 4 and accompanying legends appear below.

**Figure 3:**
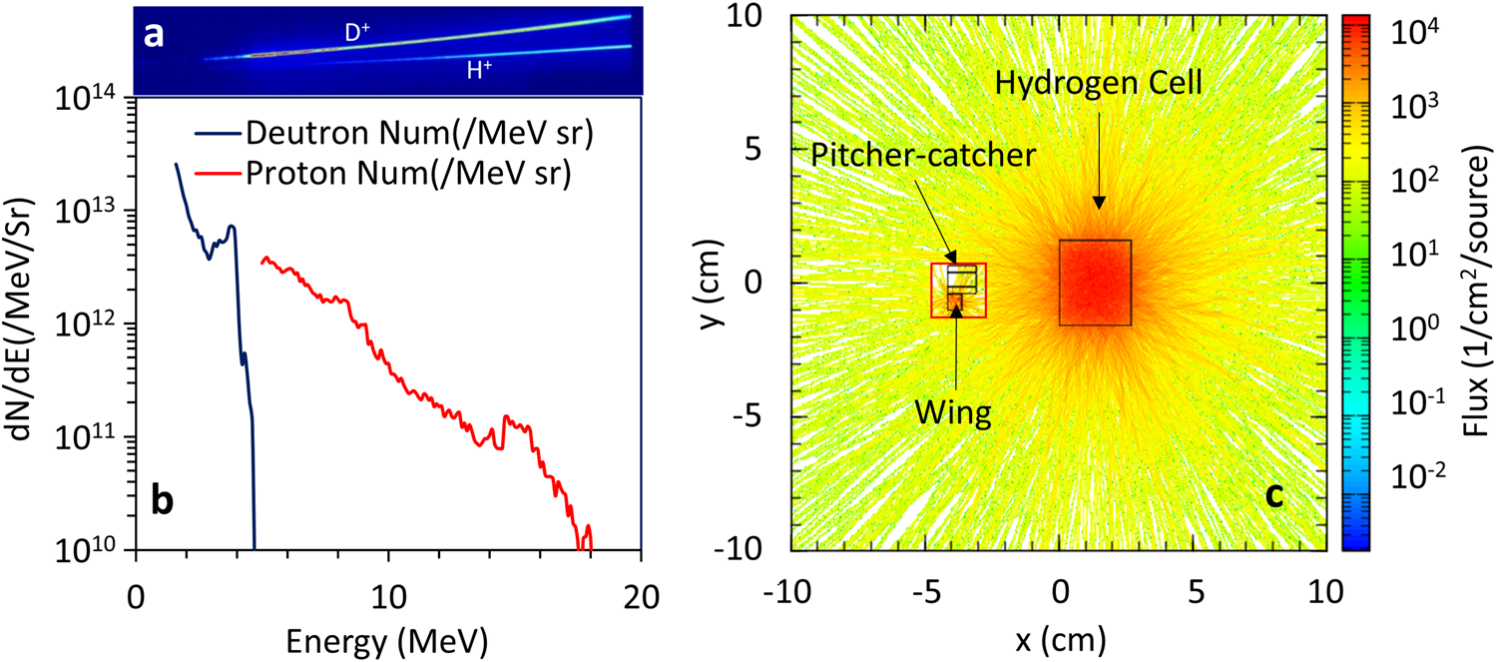
Ion-driver spectrum with corresponding neutron simulation. (**a**) Shows a typical proton and deuteron raw data obtained using 5μm5μm Au foil with TP, with respected spectra shown in (**b**). (**c**) shows the PHITS Monte-Carlo simulation performed for the cold neutrons (≤ 25 meV ≤ 25 meV) in the hydrogen moderator, confirming an isotropic nature of the moderated neutrons.

**Figure 4:**
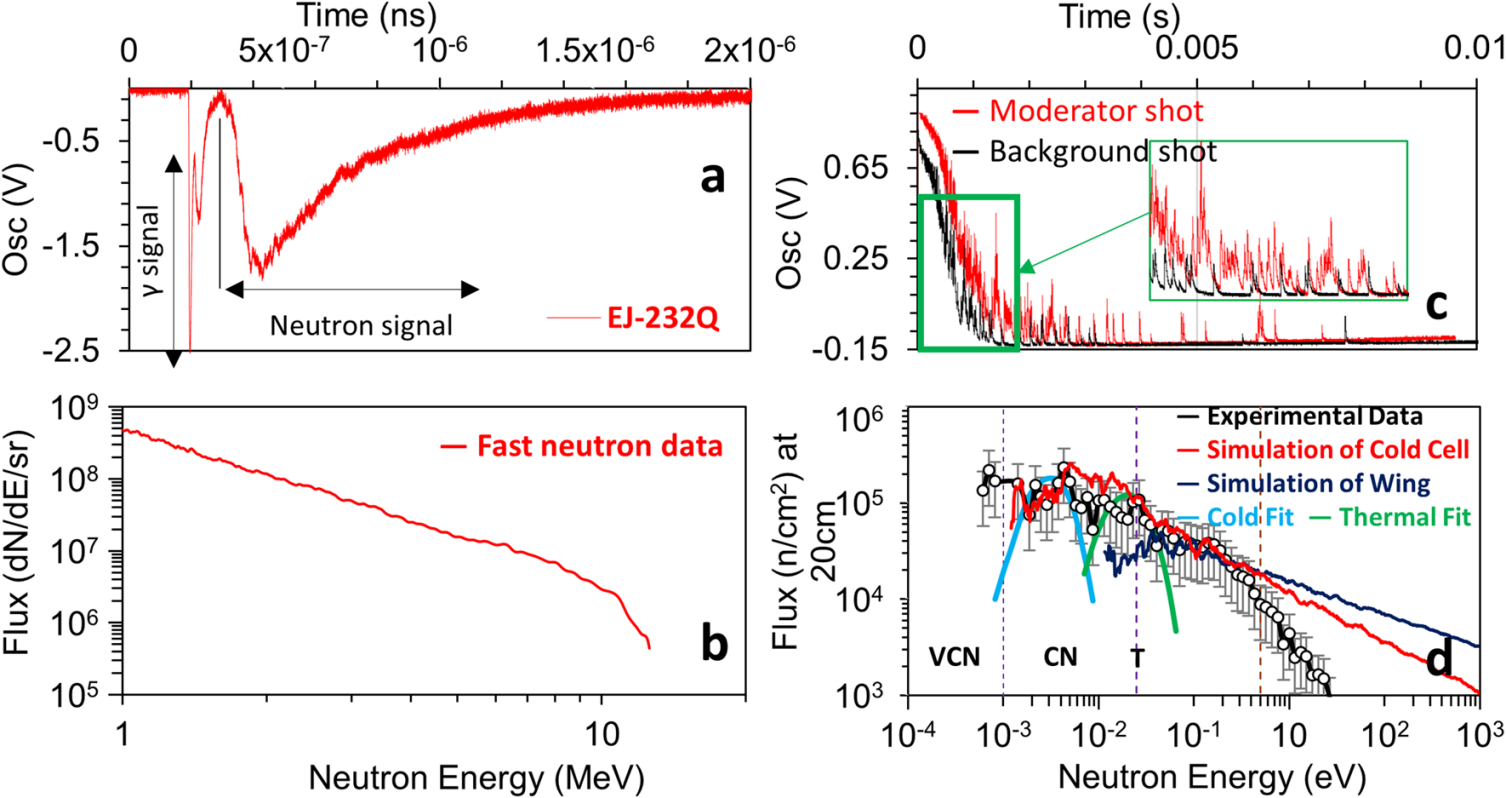
Experimental results. (**a**) is the raw signal recorded using EJ-232Q plastic scintillator and the corresponding spectrum shown in (**b**), which was calculated by taking into account the detector distance, efficiency and the transmission. (**c**) demonstrating the neutron spikes generated by nuclear reaction of 3He(n,p)3H3He(n,p)3H in the proportional counter for a typical moderator shot (with hydrogen) and no moderator shot (without hydrogen). The background-subtracted data at 20 cm exit surface of the moderator showing a good agreement with the Monte-Carlo simulation results as shown in (**d**). The broadened peak of neutrons extended its tail reaching 0.8 meV and has been confirmed using two Maxwellian numerically calculated fits for the cold and thermal temperature. The navy blue line is showing the contribution of the wing moderator which is barely reaching the thermal peak.

Finally, the Author Contributions section,

“The moderator and experiment was developed by S.R.M. with support from A.I. and funded by A.Y. to and led by S.R.M. S.R.M., A.Y., Z.L. and T.I. executed the experiment with support from, AI, KM, MNag, MNak, YAr, YAb, D.G., Y.H., T.M., S.S. and K.O. contributed both to the experimental setup and diagnostics. S.R.M. and Z.L., T.I. performed data analysis.”

now reads:

“The moderator and experiment were developed by S.R.M. with support from A.I. The experiment funded by A.Y. and led by S.R.M. S.R.M., A.Y., Z.L. and T.I. executed the experiment with support from A.I., K.M., M.Nag, M.Nak, Y.Ar., Y.Ab., D.G., Y.H., T.M., S.S. and K. contributed both to the experimental setup and diagnostics. S.R.M., Z.L. and T.I. performed the data analysis. The manuscript was prepared by S.R.M., D.N., S.F., K.M., H.N., S.K. and R.K. were involved in discussions and preparation.”

The original Article has been corrected.

